# Robot-Assisted Reinforcement with a Free Pericardial Fat Pad for Double-Lumen Tube–Related Injury of the Left Main Bronchus Incidentally Detected during Lobectomy: A Case Report

**DOI:** 10.70352/scrj.cr.25-0815

**Published:** 2026-04-23

**Authors:** Michihito Toda, Tomohiro Yasukawa, Ryuhei Morita, Aya Yamamoto, Takashi Iwata

**Affiliations:** Department of General Thoracic Surgery, Kansai Rosai Hospital, Japan Organization of Occupational Health and Safety, Amagasaki, Hyogo, Japan

**Keywords:** tracheobronchial injury, robot-assisted thoracic surgery, pericardial fat pad reinforcement

## Abstract

**INTRODUCTION:**

Tracheobronchial injury associated with double-lumen tube (DLT) placement is rare and typically diagnosed postoperatively. Intraoperative detection of subtle airway lesions is extremely uncommon. We report a case of DLT-related injury of the left main bronchus that was incidentally detected during robot-assisted thoracic surgery (RATS) and successfully reinforced with a free pericardial fat pad.

**CASE PRESENTATION:**

An 82-year-old man underwent robot-assisted left lower lobectomy and mediastinal lymph node dissection for suspected primary or metastatic lung cancer. During subcarinal lymph node dissection, a bronchial injury was identified at the distal end of the left main bronchus, at the junction between the membranous and cartilaginous portions. The bronchial wall showed a longitudinal laceration; the muscular layer was torn, but the mucosa remained barely intact. The balloon of the blue bronchial tube could be seen through the mucosa within the lumen. Bronchoscopy revealed a bronchial injury at the distal end of the left main bronchus, at the junction between the membranous and cartilaginous portions, likely caused by the DLT cuff. From the luminal side, mucosal redness and protrusion into the lumen—presumed to be caused by increased intrathoracic pressure—were observed, raising suspicion of disruption limited to the outer bronchial wall structures, such as the smooth muscle layer, with the mucosa spared. Following lobectomy, a free pericardial fat pad harvested from the anterior mediastinum was applied to the injured site with bioadhesive and sutured to be covered with mediastinal pleura. Postoperative bronchoscopy and CT revealed stable graft attachment without evidence of leakage or infection. The patient recovered uneventfully and was discharged on POD 7.

**CONCLUSIONS:**

RATS enabled early detection and safe repair of a subtle airway injury that otherwise may have been overlooked. Free adipose tissue reinforcement is a feasible option for managing DLT-related membranous thinning. This case highlights the risks of DLT-related airway injury and the safe management strategies achievable using RATS.

## Abbreviations

DLT
: double-lumen tube
RATS
: robot-assisted thoracic surgery
S#
: lung segment number

## INTRODUCTION

Tracheobronchial injury is a rare but potentially serious complication of tracheal intubation, particularly during 1-lung ventilation using a DLT.^1,2)^ Although DLTs facilitate effective lung separation, their large diameter, cuff pressure, and technical complexity can predispose patients to mucosal tears or thinning of the membranous wall.^1–6)^ Such injuries are typically diagnosed postoperatively following clinical manifestations such as subcutaneous emphysema, air leakage, or ventilatory failure, whereas intraoperative identification is exceedingly uncommon.^1–3)^

RATS provides high-definition, 3D visualization and enhanced instrument maneuverability, allowing precise dissection and real-time recognition of subtle anatomical changes. These advantages may facilitate intraoperative identification of bronchial membranous abnormalities that would otherwise remain unnoticed during conventional thoracoscopic or open surgery.^7)^

Primary repair with sutures or coverage using muscle or pericardial fat pad is commonly performed for bronchial injuries. However, surgical reinforcement of the mucosal layer–preserved bronchial incomplete injury has rarely been reported, and to our knowledge, reinforcement using a free pericardial fat pad for such injury under robotic assistance is extremely rare.^6,7)^

We report a successfully recovered case of incidentally detected DLT-related membranous thinning of the left main bronchus during robot-assisted left lower lobectomy and mediastinal lymph node dissection, which was robotically reinforced using a free pericardial fat pad.

## CASE PRESENTATION

**Fig. 1:**
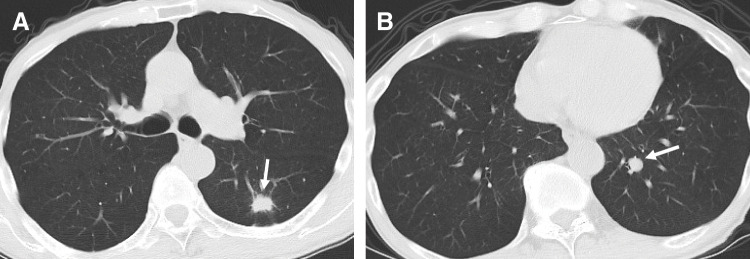
Preoperative chest CT scans. (**A**) A spiculated nodule measuring 19 × 16 mm was identified in segment 6 of the left lower lobe (arrow). (**B**) A round nodule measuring 8 × 4 mm was detected in segment 10 (arrow).

The patient was an 82-year-old man (height, 162 cm; weight, 47 kg) with an Eastern Cooperative Oncology Group performance status score of 0. He had a 62-year history of smoking 20 cigarettes per day, which he had discontinued 3 years prior. He had undergone low anterior resection for stage IIIB rectal cancer 2 months prior to the lung resection. Chest CT before rectal surgery revealed a 14-mm nodule in the left S6 segment and a 6-mm nodule in the S10 segment (**Fig. 1A** and **1B**). PET-CT demonstrated abnormal fluorodeoxyglucose uptake in both nodules, prompting suspicion of primary lung cancer or pulmonary metastases from rectal cancer. Pulmonary function tests revealed no obstructive or restrictive impairment. A preoperative pathological diagnosis was not pursued, and robot-assisted left lower lobectomy with mediastinal lymph node dissection (ND2a-1) was planned for diagnostic and therapeutic purposes.

Surgery was performed in the right lateral decubitus position under 1-lung ventilation using a left-sided DLT (peripheral cuff inflated with 3 mL; cuff pressure maintained at 20 cm H_2_O). Intubation was achieved without difficulty. The procedure was performed using the da Vinci Xi system (Intuitive Surgical, Sunnyvale, CA, USA). Extensive fibrous adhesions were observed throughout the thoracic cavity, predominantly in the upper lobe. According to the standard approach for lower lobectomy, dissection of the pulmonary ligament, posterior hilar release, fissure development, division of the pulmonary arterial branches (A6, A8, and A9 + 10), tunneling and stapling of the inferior pulmonary vein, and subsequent lymph node dissection (#7, #10, and #11) were performed.

**Fig. 2:**
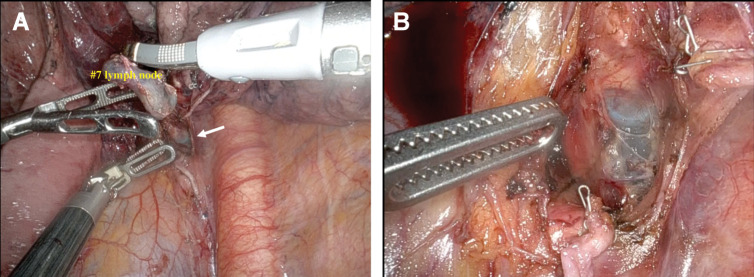
Intraoperative findings during subcarinal lymph node dissection. (**A**) The distal cuff of the DLT was visualized through the membranous portion of the left main bronchus (arrow). (**B**) Membranous thinning allowed visualization of the tube tip, although the membrane structure remained intact. DLT, double-lumen tube

**Fig. 3:**
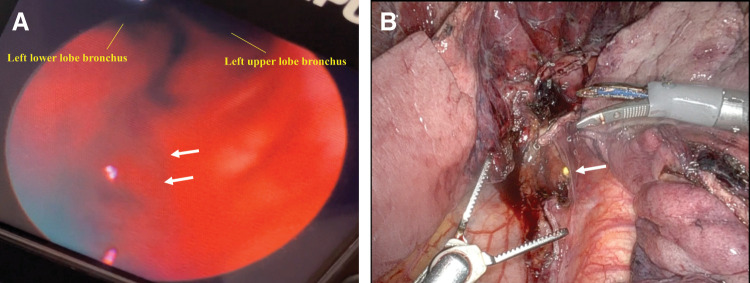
Intraoperative bronchoscopic findings. (**A**) Bronchoscopy revealed thinning of the membranous portion of the left main bronchus at the cuff contact site, just proximal to the left second carina (arrows). (**B**) The bronchoscopic light was visible from the thoracic cavity through the thinned membrane (arrow).

During subcarinal (#7) lymph node dissection, a bluish structure was visualized through the membranous portion of the left main bronchus (**Fig. 2A**), suggesting distal DLT cuff protrusion (**Fig. 2B**). Upon partial withdrawal of the tube at the direction of the anesthesiology team, the tip of the endotracheal tube became visible just beneath the membranous portion. Further withdrawal of the tube under bronchoscopic guidance confirmed that the distal cuff was in direct contact with the thinned membranous wall. Intraoperative bronchoscopy demonstrated focal thinning of the membranous tissue immediately proximal to the secondary carina, which was considered to be induced by cuff compression (**Fig. 3A** and **3B**). From the luminal side, mucosal erythema and inward protrusion were observed, presumably resulting from increased intrathoracic pressure by CO_2_ insufflation. The injury appeared to be confined to the extramucosal wall structures, including the smooth muscle layer, while the mucosa itself seemed to be preserved. The endotracheal tube was subsequently repositioned to prevent further injury. We first proceeded to complete the lobectomy. The lower lobe bronchus was clamped, and positive-pressure ventilation was applied to the left lung to confirm adequate expansion of the remaining lung. At the same time, the absence of air leakage from the injured membranous portion of the left main bronchus was confirmed, indicating that the injury had not progressed to a full-thickness disruption. The lower lobe bronchus was then divided, and the lobectomy was completed.

**Fig. 4:**
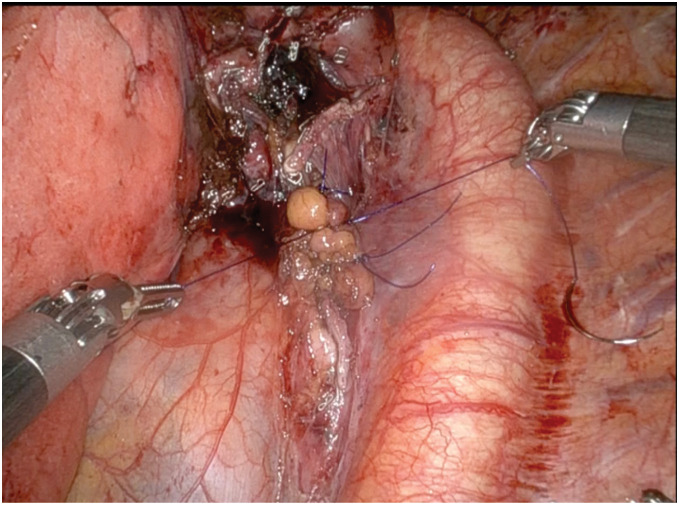
Robot-assisted adipose tissue graft application. Mobilized fat pad was sutured onto the thinned membranous portion of the left main bronchus using the robotic system.

After completing the lobectomy, reinforcement of the exposed mucosal part was performed. A free fat pad measuring approximately 3 cm was harvested from the anterior mediastinum. Bioadhesive fibrinogen solution was applied to the membranous surface, followed by placement of the fat pad and application of thrombin solution to ensure adherence. The graft was then covered with mediastinal pleura and secured using 3 interrupted 4-0 PDS II sutures (Ethicon, Somerville, NJ, USA) II sutures (**Fig. 4**). As no air leakage was detected from the injured membranous portion during bronchial clamping, and repeated positive-pressure ventilation was considered to pose a risk of aggravating the fragile membrane and causing full-thickness disruption, no additional sealing test was performed. Hemostasis was confirmed and a 28-Fr. chest drain tube (Nipro, Osaka, Japan) was placed.

**Fig. 5:**
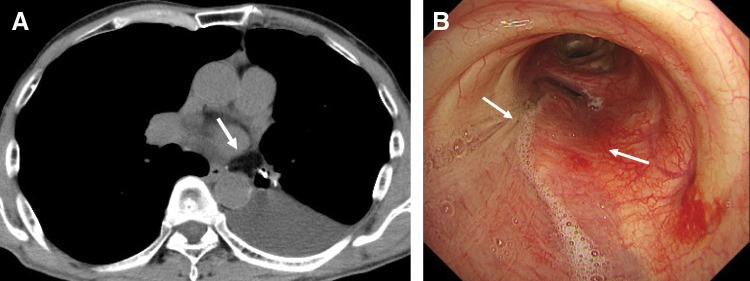
Postoperative imaging on day 6. (**A**) Chest CT shows the free fat pad adherent along the right-to-dorsal aspect of the left main bronchus and the second carina (arrow). (**B**) Bronchoscopy reveals loss of longitudinal folds at the previously thinned membranous portion of the left main bronchus, with the underlying fat pad visible through the membrane (arrows).

Operative time was 2 h 47 min (console time, 2 h 4 min) with no blood loss or transfusion requirement. The chest drain was removed on POD 1. Bronchoscopy and CT performed on POD 6 demonstrated stable graft attachment without air leakage or infection (**Fig. 5A** and **5B**). The patient was discharged on POD 7.

Pathological examination revealed primary squamous cell carcinoma (pStage IA2) in S6 and metastatic rectal adenocarcinoma in S10. No adjuvant treatment was administered.

## DISCUSSION

Tracheobronchial injury associated with DLT placement is rare but potentially life-threatening.^1–5)^ Reported risk factors include older age, female sex, short stature, fragile airway mucosa, and excessive cuff pressure.^1,3,4)^ Most cases are diagnosed postoperatively, whereas intraoperative recognition remains extremely uncommon.^1–3)^

In the present case, distal DLT cuff pressure was maintained at 20 cm H_2_O, which is generally not considered excessive. However, the patient’s lean body habitus, advanced age, and extensive adhesions to the chest wall suggested underlying airway fragility that may be related to prior inflammatory processes. Additionally, traction forces applied to the lung owing to adhesions between the left upper lobe and chest wall may have increased localized mechanical stress within the airway, thereby contributing to injury of the membranous portion.

The injury was identified after subcarinal lymph node dissection. Careful review of the operative video revealed no evidence of direct mechanical trauma to the membranous bronchial wall by surgical instruments. The location of the muscular tear corresponded precisely to the site of distal DLT cuff contact, supporting cuff-related compression as the primary mechanism. The preservation of the mucosal layer with disruption limited to the smooth muscle layer may be explained by differences in compliance between the mucosa and the muscular layer in response to compressive stress.

However, a multifactorial etiology should also be considered. In a bronchus that may have been structurally vulnerable, excessive localized pressure from the distal cuff, together with traction forces generated during subcarinal lymph node dissection—particularly at the cartilage–membrane junction—may have contributed to disruption of the smooth muscle layer.

With regard to prevention from the anesthesiologist’s perspective, as previously reported in the literature,^8,9)^ careful management of cuff pressure and tube positioning during DLT placement and patient repositioning is essential. In the present case, cuff pressure was maintained within the recommended range; however, patient-related factors such as advanced age, low body weight, and suspected airway fragility may have increased the risk of cuff-related bronchial injury.

Other reported risk factors include chronic steroid use, prior radiotherapy, local airway inflammation, tracheobronchomalacia, and anatomical deviations such as scoliosis or tumor-related airway distortion.^8–10)^ Careful bronchoscopic confirmation of tube position, frequent intraoperative reassessment, and individualized cuff pressure management according to patient characteristics are therefore crucial.

Furthermore, particularly in procedures involving extensive traction or subcarinal lymph node dissection, close intraoperative communication between the anesthesiologist and the surgeon is indispensable to allow timely adjustment of tube position and cuff inflation.

Subtle membranous injury was identifiable because of the high-resolution, 3D visualization provided by RATS. Previous reports have similarly described successful intraoperative detection and robot-assisted repair of airway injuries.^7)^ Without the enhanced visualization by the robotic platform, the lesion might have gone unrecognized and could have progressed to a full-thickness rupture postoperatively.

Repair strategies for bronchial injuries vary depending on lesion depth and extent, and include primary suture repair, muscle or pericardial flap coverage, and sealant application^1,6)^. In recent years, several case reports have described favorable outcomes following robot-assisted primary suture closure of tracheal or bronchial injuries, including membranous lesions, without conversion to thoracotomy.^7,11)^ These reports demonstrate that the robotic platform—through its high-definition 3D visualization, tremor filtration, and superior instrument dexterity—enables precise suturing even in anatomically complex airway regions.

Direct suture repair is generally recommended for intraoperative tracheobronchial injuries, including those caused by intubation, as described by Welter.^12)^ However, in cases limited to incomplete injury without progression to full-thickness disruption, such as in our presented case, direct suture closure may not necessarily represent the optimal treatment strategy.

Cardillo et al. proposed a morphological classification of postintubation tracheobronchial lacerations based on lesion depth and associated complications, derived from an analysis of 30 patients with iatrogenic tracheal injuries.^13)^ In this classification, level II injuries are defined as partial-thickness lacerations involving the muscular layer without complete transmural disruption or associated mediastinal emphysema or esophageal involvement. For these lesions, favorable outcomes were reported with nonsurgical management, including conservative observation and local coverage using fibrin sealants or similar materials, without progression to airway rupture or mortality.

According to this classification, our case corresponds to a level II injury, characterized by disruption of the extramucosal structures with preserved mucosal continuity and maintained bronchial integrity. In such situations, reinforcement of the weakened bronchial wall rather than direct suturing may represent a more appropriate strategy to avoid additional tissue damage.

In the present case, incomplete injury of the transitional area between the membranous and cartilaginous parts of the left main bronchus caused by distal cuff pressure of the DLT was identified, while mucosal continuity was preserved. Therefore, reinforcement with a free pericardial fat pad was selected instead of direct suturing. Pericardial fat provides sufficient volume, excellent flexibility and conformity, allowing it to function as a biological patch that covers the weakened membranous area and protects the lesion from mechanical stress induced by positive-pressure ventilation and postoperative coughing.^14)^ In addition, fat tissue promotes healing through passive imbibition and subsequent neovascularization from surrounding tissues, without requiring tension-bearing sutures, which represents a further advantage in the management of fragile airway lesions.^15)^ Another advantage includes the ease of harvesting and suturing within the operative field, enabling a minimally invasive repair of mild-to-moderate bronchial injuries without requiring muscle or pleural flaps. In the present case, the graft was safely and reliably applied using RATS.

Overall, RATS played a pivotal role in this case by enabling both early detection of a subtle airway lesion and precise, minimally invasive reinforcement with a free fat pad. To the best of our knowledge, reports describing robot-assisted free fat pad reinforcement for incomplete bronchial injury remain extremely limited. This case suggests that, in level I–II tracheal or bronchial injuries with preserved structural continuity, robot-assisted fat pad reinforcement may represent a safe, feasible, and practical therapeutic option, while also highlighting the potential risks of DLT-related airway injury and the advantages of robotic surgery in achieving safe intraoperative management.

## CONCLUSIONS

RATS can facilitate early detection and safe repair of subtle DLT-related airway injuries. Free fat pad reinforcement is a feasible and minimally invasive option for managing membranous thinning when bronchial structural integrity is preserved.
